# *Beta vulgaris* Assisted Fabrication of Novel Ag-Cu Bimetallic Nanoparticles for Growth Inhibition and Virulence in *Candida albicans*

**DOI:** 10.3390/pharmaceutics13111957

**Published:** 2021-11-18

**Authors:** Majid Rasool Kamli, Maqsood Ahmad Malik, Shabir Ahmad Lone, Jamal S. M. Sabir, Ehab H. Mattar, Aijaz Ahmad

**Affiliations:** 1 Department of Biological Sciences, Faculty of Sciences, King Abdulaziz University, P.O. Box 80203, Jeddah 21589, Saudi Arabia; jsabir2622@gmail.com (J.S.M.S.); emattar@kau.edu.sa (E.H.M.); 2 Center of Excellence in Bionanoscience Research, King Abdulaziz University, P.O. Box 80203, Jeddah 21589, Saudi Arabia; 3 Chemistry Department, Faculty of Sciences, King Abdulaziz University, P.O. Box 80203, Jeddah 21589, Saudi Arabia; 4 Clinical Microbiology and Infectious Diseases, Faculty of Health Sciences, School of Pathology, University of the Witwatersrand, Johannesburg 2193, South Africa; shabir.lone@wits.ac.za (S.A.L.); Aijaz.Ahmad@wits.ac.za (A.A.); 5 Infection Control Unit, Charlotte Maxeke Johannesburg Academic Hospital, National Health Laboratory Service, Johannesburg 2193, South Africa

**Keywords:** bimetallic nanoparticle, *Candida albicans*, virulence factors, biofilm, cell viability

## Abstract

*Beta vulgaris* extract contains water-soluble red pigment betanin and is used as a food colorant. In this study, the biogenic Ag-Cu bimetallic nanoparticles were synthesized and characterized by different spectroscopic and microscopic techniques, including UV–Visible, FTIR, TEM. SEM-EDX, XRD, and TGA. Further, Ag-Cu bimetallic nanoparticles capped with *Beta vulgaris* biomolecules were evaluated for their antifungal activity against *Candida albicans* via targeting its major virulence factors, including adherence, yeast to hyphae transition, extracellular enzyme secretion, biofilm formation, and the expression of genes related to these pathogenic traits by using standard methods. *C. albicans* is an opportunistic human fungal pathogen that causes significant morbidity and mortality, mainly in immunocompromised patients. The current antifungal therapy is limited with various shortcomings such as host toxicity and developing multidrug resistance. Therefore, the development of novel antifungal agents is urgently required. Furthermore, NPs were screened for cell viability and cytotoxicity effect. Antifungal susceptibility testing showed potent antifungal activity of the Ag-Cu bimetallic NPs with a significant inhibitory effect on adherence, yeast to hyphae transition, extracellular enzymes secretion, and formation of biofilms in *C. albicans* at sub-inhibitory and inhibitory concentrations. The RT-qPCR results at an MIC value of the NPs exhibited a varying degree of downregulation in expression levels of virulence genes. Results also revealed the dose-dependent effect of NPs on cellular viability (up to 100%) using MUSE cell analyzer. Moreover, the low cytotoxicity effect of bimetallic NPs has been observed using haemolytic assay. The overall results indicated that the newly synthesized Ag-Cu bimetallic NPs capped with *Beta vulgaris* are proven to possess a potent anticandidal activity, by affecting the vital pathogenic factors of *C. albicans*.

## 1. Introduction

*Beta vulgaris* is a rich source of water-soluble nitrogenous pigments known as betalains, classified into red-violet (betacyanins) and yellow (betaxanthins) pigments that have long been used natural colorants in food. Betacyanins, primarily betanin and its isomers, make up most of the pigments in red beetroot [1,2,3]. Because they are non-toxic, good stabilizing agents and a rich supply of vitamins, proteins, polyphenols, carbohydrates, and dyes, extracts derived from natural products have been widely used to fabricate many types of nanoparticles [4,5,6,7]. In addition, natural dyes are well-known for being non-allergic, biodegradable, and safe reducing agents [8]. The development of the bimetallic nanoparticles is advanced, based on their synergic properties of two participating metal salts in a mixture solution [9,10]. Bimetallic nanoparticles are preferred because they acquire much better optical, magnetic, electronic, catalytic, and antioxidant properties than metals [11,12,13,14]. These bimetallic Ag-Cu NPs possess properties of high electron conductivity, optical properties and are utilized as active antibacterial agents [15,16,17,18,19]. Consequently, the present work relies on the concept that the chemical stability of Cu is being improved upon the fabrication of Ag onto the surface of Cu to delay the release of Cu^2+^ ions [20,21]. Thus, the nano-assembly was expected to be an efficient candidate against *C. albicans* an opportunistic human fungal pathogen. The metallic nanoparticles are being developed by using various physical, chemical, and green methods [22]. Although, the “Green synthesis” using biomass such as plant parts, bacteria, and fungi in the biogenesis of bimetallic nanoparticles [23]. The use of a greener route for nanomaterial synthesis has received a lot of attention as a sustainable, feasible, dependable, cost-effective, wealthy, and environmentally friendly architype [24]. Greener synthesis is thought to be a sophisticated tool for minimizing the hazardous consequences associated with traditional synthesis methods created for nanomaterials that are typically preferred in industry and laboratories [24]. In the primary research, these biomolecules and organisms can identify inorganic surfaces and are utilized as the surface of matrices to synthesize inorganic nanoparticles. However, the biogenic method of synthesis of nanoparticles has been found to be advanced in comparison to other synthetic approaches; phytochemicals are being utilized as reducing/capping agents [25]. These methods are effective in certain conditions and depend on extraction solvent, solvent concentration, plant parts, pH of the reaction mixture, and salt concentration [5]. Moreover, *Beta vulgaris* L. has a sugar-rich composition and possesses an efficient reductive capability to synthesize nanomaterials [26,27]. For example, *Beta vulgaris* L. extract, a rich source of pigment betanin, was used in a single-pot biosynthesis of Ca-based nanoparticles [4]. In addition, the phytochemical composition of *Beta vulgaris* L. extract was used as a reducing/capping agent in the biosynthesis of Ag NPs, which showed enhanced catalytic activities in the reduction of 4-nitrophenol to 4-aminophenol [6].

Fungi have been implicated as causative agents for human diseases, particularly in immunodepleting patients such as HIV and cancer patients, transplant recipients, diabetes patients, premature babies, and any patients who stayed in the hospital for a long time. Among fungal pathogens, *Candida* species are the most frequently reported opportunistic fungal pathogen in humans causing mucosal infections [28] and are at number four in causing nosocomial infections [29]. Furthermore, *Candida* infections have also been recognized as a common cause of septicemia in neonates with high mortality and morbidity (25–54%) [30]. Despite the high rise of non-albicans *Candida* infections, *C. albicans* is still leading, accounting for causing around 90% of invasive infections around the globe [31]. Triazoles and polyenes are the first choices used in clinics to treat *Candida* infections; however, the use and abuse of these limited antifungal drugs have resulted in the emergence of high drug-tolerant clinical isolates of *C. albicans*. The drug resistance problem in *Candida* is further amplified due to the restricted drug targets of these antifungals. This has significantly increased the need to develop new antifungal drugs with different mechanisms of action. One novel strategy to develop new antifungal drugs targets virulence factors and biofilm formation [32]. Being an opportunistic pathogen, *C. albicans* are reported to possess different virulence factors such as adherence, morphological transition, and secretion of hydrolytic enzymes to transform from normal commensal to pathogenic forms [33]. Adherence and morphological change from yeast to hyphae forms are the initial steps in the pathogenicity of *C. albicans* and are reported to possess characteristics as an emerging drug target [32]. The pathogenicity in *C. albicans* is supported by extracellular hydrolytic enzymes (proteinases and phospholipases). They are directly involved in yeast overgrowth, enabling adherence, host tissue penetration, and subsequently host tissue invasion [34]. Another unavoidable factor is biofilms formed on abiotic and/or biotic surfaces, which are widely believed to possess high resistance to most commonly used antifungal drugs, are sessile cells [35]. As a result of the production of different hydrolytic enzymes, morphogenesis, and formation of drug-resistant biofilms in *C. albicans*, there is an urgent need for innovative strategies to develop new and safer antifungal agents targeting various virulence factors in *C. albicans* and successfully eradicating life-threatening infections caused by this pathogenic yeast. In the current study, novel Ag-Cu NPs were synthesized and tested for anti-candidal activity. Furthermore, the Ag-Cu bimetallic NPs were further investigated for inhibiting crucial pathogenic attributes in *C. albicans*. Additionally, these NPs were also evaluated for their cytotoxic activity using horse red-blood cells.

## 2. Materials and Methods

*Beta vulgaris,* a beetroot, was collected from the local market in Jeddah, Saudi Arabia. Copper nitrate (Cu(NO_3_)_2_·3H_2_O, 99.5% pure), Silver nitrate (AgNO_3_, 99.5% pure), ethanol (C_2_H_6_O, purity ≥ 99.9%) were purchased from Merck, Darmstadt, Germany, and used directly without further treatment. Deionized water was used throughout the work for the preparation of the precursor solutions and *Beta vulgaris* extract.

### 2.1. Design and Preparation of Ag-Cu NPs

*Beta vulgaris* was initially washed thoroughly under Tap water and then distilled water to remove all dirt from the surface. The *Beta vulgaris* (10 g) was pulverized into small pieces and ground using a blender. The grounded *Beta vulgaris* was mixed with 250 mL of deionized water and brought to heating for 2 h under constant stirring. The extract was cleaned using Whatman filter paper No 1 and stored in an amber color glass container in the dark to prevent betanin decomposition. The green synthesis of Ag-Cu bimetallic nanoparticles was carried out in a one-pot system by mixing 50 mL of Ag^+^ and Cu^2+^ ion solution in a 1:1 molar ratio. The reaction mixture was constantly stirred at 30 °C followed by 50 mL *Beta vulgaris* aqueous extract. The formation of Ag-Cu bimetallic nanoparticles was visualized by the color change from dark pinkish to dark brown within a few minutes, and the reaction was completed within 24 h. The reaction mixture was constantly stirred at 30 °C and finally centrifuged at 10,000× *g* rpm for 30 min to collect the nanoparticles from the supernatant. The as-prepared nanoparticles were dried in an oven at 80 °C for 24 h and stored. Microscopic and spectrophotometric techniques further characterized the dried samples.

### 2.2. Characterization

The surface morphology, size, and elemental composition of the synthesized Ag-Cu nanoparticles were determined using several characterization techniques. To ascertain the absorption spectroscopy of range between 200–800 nm, a UV–Vis spectra was recorded using a double beam spectrophotometer (Shimadzu UV–Vis MultiSpec-1501, Osaka, Japan). Meanwhile, the biosynthesis was further established by presence of functional groups onto the surface of as-prepared nanoparticles using a PerkinElmer FT-IR spectrometer (Waltham, MA, USA) with a scan range of 450–4000 cm^−1^. Furthermore, a BRUKER D8 ADVANCE X-ray diffractometer (XRD) (Bruker AXS GmbH, Karlsruhe, Germany) with Cu-Kα (1.54184) was utilized to conclude the particles diffraction pattern and crystalline characteristics by means of X-ray diffraction. The samples’ shape and size distribution were determined using transmission electron microscopy (TEM) (JEM-3010, JEOL, Tokyo, Japan) studies at 200 kV. The scanning electron microscopy (SEM) (JEOL JSM-7600F) technique was used to create sample images and understand the particle morphology. Finally, the elemental composition of the produced nanoparticles was determined using energy-dispersive X-ray spectroscopy (EDX) at an accelerating voltage of 20kV. Additionally, ImageJ software (LOCI, University of Wisconsin, Madison, WI, USA) was used to analyze 2D TEM pictures, first by adjusting the scale shown on the images and then using the analysis tool inside the software to quantify the NPs for particle size distribution. The thermogravimetric study was performed on the dried powder of bio-synthesized Ag-CuNPs. The TGA was performed on an SDT Q600 analyzer (TA Instruments, New Castle, DE, USA) in a nitrogen environment with a heating rate of 10°/min and a temperature range of 30–800 °C.

### 2.3. Strains and Media

In this study 9 isolates of *C. albicans*, including 4 clinical fluconazole (FLZ) susceptible strains (4554, 4251, 4175, and 4180) and 4 clinical FLZ resistant strains (4324, 4106, 5112, and 4085) and a *C. albicans* SC5314 (laboratory control strain) were used. All the clinical isolates were obtained from Charlotte Maxeke Johannesburg Academic Hospital under ethical clearance number M000402. The ethical clearance was issued from the HREC, Wits University, Johannesburg. All the strains were stored in the department at −80 °C as glycerol stocks and were subculture on Sabouraud Dextrose agar (SDA) before use.

### 2.4. Antifungal Susceptibility Testing

Minimum Inhibitory Concentration (MIC) and Minimum Fungicidal Concentration (MFC) values of Ag-Cu bimetallic NPs against various *C. albicans* strains were estimated by the broth microdilution method following Clinical and Laboratory Standards Institute (CLSI 2010) guidelines M27A [36]. An amount of 1% dimethyl sulfoxide (DMSO) was used to prepare the NP and FLC stock solutions. In each experiment, positive (FLC), negative (1% DMSO), culture (media and cells only), and sterility (media only) controls were included. The lowest concentrations of the NPs with no visible growth were considered as the MIC value.

For MFC values, each well without growth was cultured onto the SDA plates and incubated for 24 h at 37 °C. The first well with no growth on the agar plate after incubation was calculated as MFC.

### 2.5. Effect of Ag-Cu Bimetallic NPs on C. albicans Cell Viability

To determine the cell viability, Muse™ Cell Analyzer (EMD Millipore, Temecula, CA, USA) was used as marked by the manufacturer. Briefly, *C. albicans* cells were exposed to ¼ × MIC, ½ × MIC and MIC of bimetallic NPs at 37 °C for 3 h. Post-incubation cells were washed, centrifuged, and then cell suspension (1 × 10^7^ cells/mL) in fresh saline was prepared. Later, the cells were stained with Muse Count and Viability kit (EMD Millipore, Temecula, CA, USA) and analyzed for their viability profile as reported earlier [37]. Negative (Unexposed cells) and positive (heat-killed cells) controls were used in every set of experiments.

### 2.6. Effect of Ag-Cu Bimetallic NPs on C. albicans Adherence to Polystyrene Surface

Adherence assay was undertaken as reported in the previous study [37]. Briefly, pre-grown *C. albicans* SC5314 cells (100 μL; 1 × 10^6^ cells/mL) were dispensed into designated wells of 96-well microtiter plate. The cells were exposed to different subinhibitory concentrations of NPs (¼ and ½-MICs) and plates were incubated at 37 °C, 3 h and without shaking. Thereafter, non-adherent cells were aspirated, and wells were gently washed with sterile PBS followed by addition of 100 μL of 5.0% alamarBlue (Thermo Fisher Scientific, Eugene, OR, USA) and further incubated for 2 h at 37 °C. The fluorescence was recorded at 555Ex/585Em using a SpectraMax iD3 multi-mode microplate reader (Molecular Devices, Sunnyvale CA, USA). In each experiment, unexposed *C. albicans* SC5314 cells were used as negative control.

### 2.7. Effect of Ag-Cu Bimetallic NPs on Yeast to Hyphal Transition

The effect of bimetallic NPs on *C. albicans* SC5314 morphogenesis was determined according to the previously reported method [38]. Briefly, the yeast cells from agar slants were grown in SDB (SigmaAldrich, St. Louis, MO, USA) at 37 °C until the late log phase was achieved. Subsequently, with the help of phosphate-buffered saline (PBS), cells were washed twice and transferred into fresh SDB, and re-incubated further for 48 h at 37 °C resulting in a synchronized yeast population. For initiation of yeast to hyphae transition, the synchronized yeast cells (50 μL) were added to a fresh SDB (10 mL) medium with 10% fetal bovine serum (SigmaAldrich, St. Louis, MO, USA). To this mixture, bimetallic NPs at desired concentrations (¼ × MIC and ½ × MIC) were added and incubated at 37 °C. The external pH of the medium was kept constant at pH 6.5. The cell aliquots were collected at different time intervals, and the morphological changes were visualized using a microscope (Leica Microsystems, Heerbrugg, Switzerland). Untreated yeast cells were also included in the study as a control.

### 2.8. Effect of NPs on C. albicans Biofilm Formation

As described earlier, the biofilm formation in untreated (control) and Ag-Cu bimetallic NPs treated *C. albicans* cells was estimated [37]. In brief: freshly grown yeast cells were inoculated (1 × 10^6^ cells/mL) in 96-well plates and incubated for 2 h at 37 °C without shaking. Post-incubation period, the media was removed, and wells were two times washed with fresh PBS followed by addition of sterile growth medium containing desired concentrations of NPs (¼ × MIC and ½ × MIC) and incubation at 37 °C for 24 h and 48 h. The outcome of NPs on *C. albicans* biofilm formation was estimated by semi-quantitative 2,3-Bis(2-methoxy-4-nitro-5-sulfo-phenyl)-2H-tetrazolium-5-carboxanilide (XTT (Invitrogen, Thermo Fisher Scientific, Eugene, OR, USA) reduction assay. For this assay, 1 mg/mL XTT and 0.4 mM menadione (SigmaAldrich, St. Louis, MO, USA) were prepared in fresh PBS and acetone, respectively. Afterward, a freshly prepared solution of XTT:menadione (20:1) was prepared and added to PBS washed pre-formed biofilms and kept in the dark for 3 h at 37 °C. Next, the wells were aspirated, and the colorimetric changes were recorded at 490 nm using SpectraMax iD3 spectrophotometer (Molecular Devices, Sunnyvale, CA, USA). The XTT reduction was directly proportional to the metabolic activity of cells embedded in *C. albicans* biofilms.

### 2.9. Confocal Studies to Evaluate the Effect of Ag-Cu Bimetallic NPs on Mature C. albicans Biofilms

To calculate the effect of Ag-Cu bimetallic NPs over 24 h and 48 h mature *C. albicans* biofilms confocal laser scanning microscopy (CLSM) study was undertaken [39]. *C. albicans* SC5314 cells were grown on glass coverslips and incubated at 37 °C for two different time intervals (24 h and 48 h). Post-incubation planktonic cells were removed, and the wells were gently washed with PBS. Next, Ag-Cu NPs at various concentrations (MIC and 2 × MIC) were dispensed in predefined wells, except for the negative control and again incubated at 37 °C for 24 h. After that, wells were rewashed, and biofilm-coated coverslips were stained (45 min, 37 °C in the dark) with fluorescent dyes FUN-1 and concanavalin A–Alexa Fluor 488 conjugate. Later, the glass coverslips bearing biofilms were observed using a fluorescence microscope (Zeiss 780, Carl Zeiss, Jena, Germany). Multitrack mode differentiates green (Concanavalin A (ConA); excitation = 488 nm and emission = 505 nm) and red (FUN-1; excitation = 543 nm and emission = 560 nm) fluorescence simultaneously.

### 2.10. Effect of Ag-Cu Bimetallic NPs on the Secretion of Extracellular Proteinases in C. albicans

The effect of NPs on the proteinase secretion was determined, as described in previous study [38]. Briefly, yeast cells were propagated in SDB (37 °C; 18 h), followed by centrifugation (3000× *g* rpm for 5 min) and washing two times with saline and resuspending yeast cells (1 × 10^6^ CFU/mL) in fresh SDB medium supplemented with different concentrations of bimetallic NPs (¼×MIC and ½×MIC) and further incubated for 3 h at 37 °C. Unexposed yeast cells were also included in the study. Post-exposure aliquots (2 µL) were spotted on proteinase agar plates, incubated at 37 °C for 4 days followed by an examination for halo zones, which determine the proteinase activity. To calculate the proteinase activity, the colony diameter by the zone of protein degradation was determined in mm.

### 2.11. Effect of Ag-Cu Bimetallic NPs on the Secretion of Extracellular Phospholipases in C. albicans

Phospholipase activity assay was performed, according to the previously reported method [38]. Yeast cells were prepared as mentioned above and exposed to varying concentrations of bimetallic NPs (¼ × MIC and ½ × MIC) for 3 h at 37 °C, whereas unexposed cells served as a control for the experiment. Next, aliquots (2 µL) were spotted at equidistant points on phospholipase agar plates and were incubated for 4 days at 37 °C. Phospholipase activity was determined by calculating the ratio of the diameter of the colony to the diameter of the precipitation zone in mm.

### 2.12. Effect of Ag-Cu Bimetallic NPs on Gene Expression in C. albicans

Real-time PCR was used to study the effect of bimetallic NPs on the virulence factor-related genes (Table S1, see Supplementary Materials) in *C. albicans* SC5314 as previously reported [37]. In brief, the total RNA extraction was undertaken using Quick–RNA Miniprep Kit (Zymo Research, Irvine, CA, USA) followed by cDNA synthesis using a iScriptTM cDNA synthesis kit (Bio-Rad, Hercules, CA, USA). Following cDNA synthesis, RT-qPCR was performed using Roche Real-time PCR system (Basel, Switzerland), and fold changes in expression were calculated using the formula 2^−(ΔΔCt)^. Primer sequences, cycling conditions, and master mix for all the genes were adopted from previous study, and the forward and reverse primer sequences are reported as supplementary data (Table S1, see Supplementary Materials) [37].

### 2.13. Cytotoxicity Studies

Haemolytic assay was performed on horse red-blood cells (National Health Laboratory Services, (NHLS), Johannesburg, South Africa) to evaluate the cytotoxic effect of Ag-Cu bimetallic NPs using previously reported method [37]. Recommended RBC suspension were treated ¼ × MIC, ½ × MIC and MIC of NP for 1 h at 37 °C. After incubation, samples were spinned for 10 min, and the absorbance of the supernatant was measured at 450 nm. 1% of Triton X-100 (Invitrogen, Thermo Fisher Scientific, Eugene, OR, USA) was used as a positive control, whereas PBS treated cells as a negative control. The following equation was applied to calculate the percentage of haemolysis:

(1) $$\%\;\mathrm{haemolysis}=\left[\frac{A450\;\mathrm{treated}\;\mathrm{sample}-\;A450\;\mathrm{unreated}\;\mathrm{sample}}{A450\;\mathrm{Triton}\;X-100\;\mathrm{treated}\;\mathrm{sample}\;–\;A450\;\mathrm{untreated}\;\mathrm{sample}}\right]\times 100\%$$

### 2.14. Statistical Analysis

The results reported were the mean of three independent readings ± standard deviation. The statistical analysis was performed by Prism 8.0.1 software (GraphPad Software, San Diego, CA, USA) using one-way ANOVA followed by Dunnett’s multiple comparisons. The *p*-value **** *p* < 0.0001 was considered as statistically significant.

## 3. Results and Discussion

**Figure 1:**
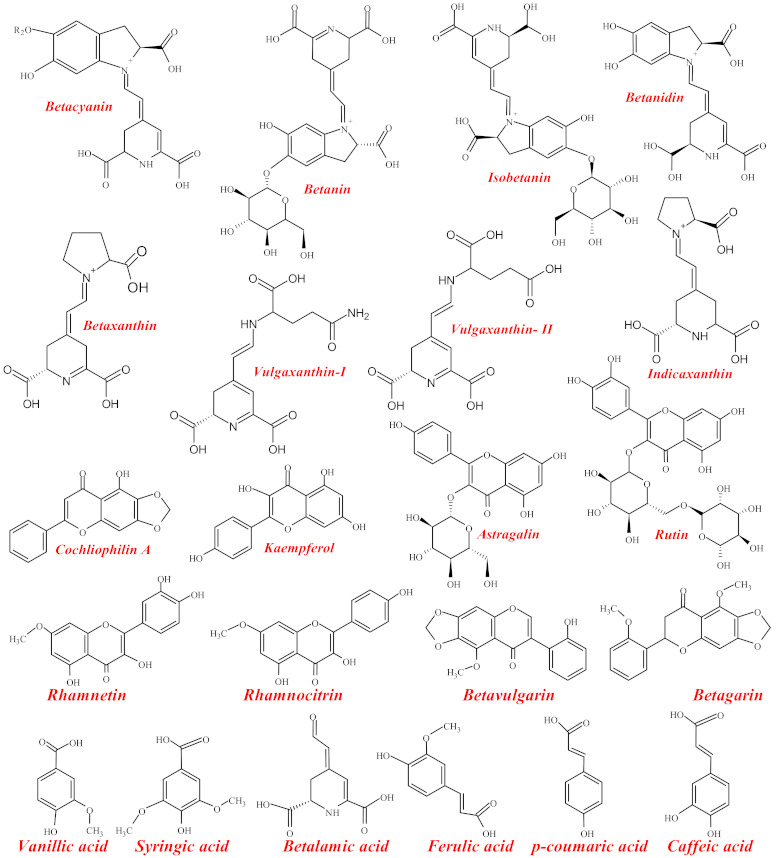
The systematic diagram is showing major phytochemicals present in the *Beta vulgaris* extract.

*Beta vulgaris* is an edible plant with numerous health benefits due to the presence of various essential biomolecules such as phenolics, carotenoids, minerals, vitamins, ascorbic acids and betalians (betacyanin-red violet pigment and betaxanthin-yellow orange pigment) [40]. The water-soluble pigments betalians (betacyanin and betaxanthin) are commercially anticipated as non-toxic, non-carcinogenic and non-poisonous food dyes [40]. *Beta vulgaris* contains highly active pigments, betalains, scorbic acid, carotenoids, polyphenols, flavonoids, saponins and high levels of nitrate. Most common bioactive phytochemicals identified in the *Beta vulgaris* are shown in Figure 1 [41,42,43]. In addition, betalains are rich nitrogenous pigments with heterocyclic rings providing colors to plant parts such as those of chlorophylls, carotenoids, anthocyanins, and other flavonoids [44]. The *Beta* *vulgaris* polyphenols and carotenoids have antioxidant, anti-inflammatory, anticarcinogenic, antidiabetic, wound healing properties, cardiovascular disease-depressing properties, hepato-protective activities, and hypertensive healing support [45,46,47].

**Figure 2:**
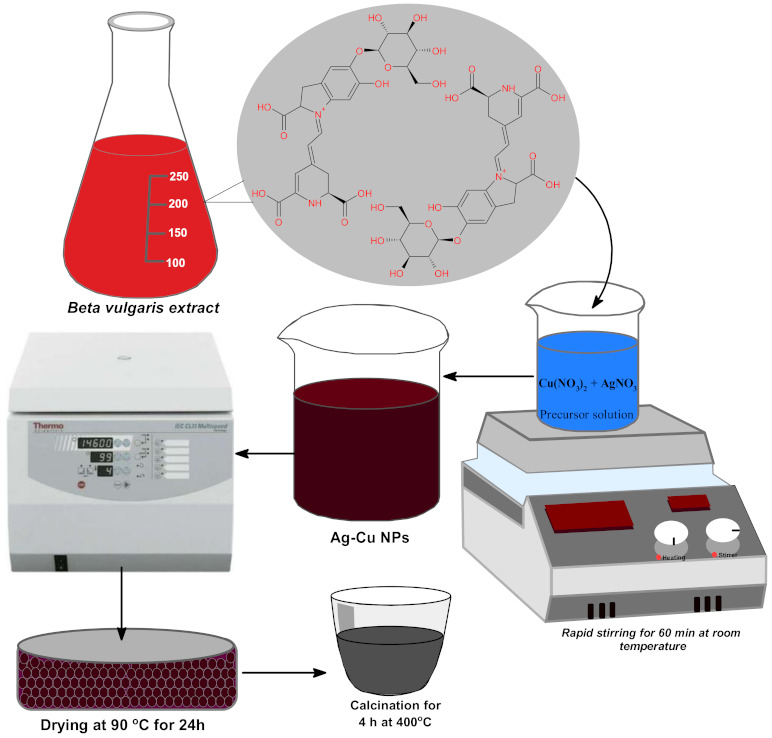
Schematic representation of *Beta* *vulgaris* assisted synthesis of Ag-Cu bimetallic nanoparticles.

The formation of bimetallic Ag-Cu nanoparticles using aqueous extract of *Beta vulgaris* is monitored by visual observations for color change from light pinkish-blue to dark brown and the schematic representation of nanoparticle formation is shown in Figure 2. The *Beta vulgaris* extract biomolecules, particularly the water-soluble nitrogenous compounds, betalains (betacyanin and betaxanthin) and betanin, are believed to possess reducing and capping properties [48,49,50]. The synthesis of Ag-Cu nanoalloy, using *Beta vulgaris* extract was established upon spectroscopic techniques, and the role played by various functional groups involved was confirmed by FTIR spectrum. The biosynthesized Ag-Cu nanoalloy was implemented in evaluation of cytotoxicity against various bacteria and fungi; herein, we investigate virulence against fungi, i.e., *C. albicans*.

**Scheme 1:**
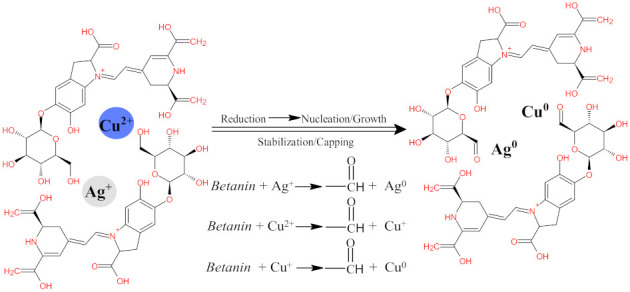
Proposed mechanism of Ag-Cu bimetallic nanoparticle formation using *Beta* *vulgaris* aqueous extract.

It is well established that the different plant parts including leaves, flowers, seeds, and fruits are rich in polyphenols, sugars, phenolic acids, alkaloids, proteins and terpenoids, which play a protuberant role in the bio-reduction of metal precursors into metal and metal oxide nanostructured materials. The one-pot biosynthesis method was developed for the synthesis of Ag-Cu bimetallic nanoparticles. Biosynthesis of nanoparticles can occur by the interaction of biomolecules of plant extract with metal precursors, which results in the bio-reduction of these precursors, in turn, resulting in the formation of stable nanoparticles, and the oxidization of biomolecules, simultaneously. The Ag^0^ and Cu^0^ were formed during the reduction of Ag^+^ and Cu^2+^ by glucose primary –OH group of betanin, as the primary -OH of glucose is very reactive as compared to the secondary –OH groups [51,52]. The mechanism of nanoparticle formation takes place in different steps, including reduction, nucleation/growth, and stabilization/capping, as shown in Scheme 1. The phytochemicals present in the extract play the role of bio-reducing agents as well as stabilizing agents. The standard reduction potential of Cu^2+^/Cu^0^ (+0.34 eV) is comparatively low as compared to the standard reduction potential of Ag^+^/Ag^0^ (0.78 eV). Consequently, the reduction of Ag^+^ occurs more rapidly than that of Cu^2+^ when mixtures of AgNO_3_ and Cu(NO_2_)_2_ were reduced by phytochemicals present in the aqueous extract of *Beta vulgaris*. However, it was observed that the bio-reduction of Cu^2+^ to Cu^0^ was enhanced due to the presence of silver ions (Ag^+^), resulting in the formation of Ag-Cu alloy nanoparticles. Therefore, it is concluded that the silver nanoparticle surface plays an essential role in the Cu^2+^ reduction enhancement. The increase in Cu^2+^ reduction in the presence of Ag particles is most likely due to the cathodic polarization of Ag-rich Ag/Cu alloy nanoparticles.

### 3.1. Beta vulgaris L. Assisted UV–Vis Spectrum of Biosynthesized Ag-Cu Nanoalloy

**Figure 3:**
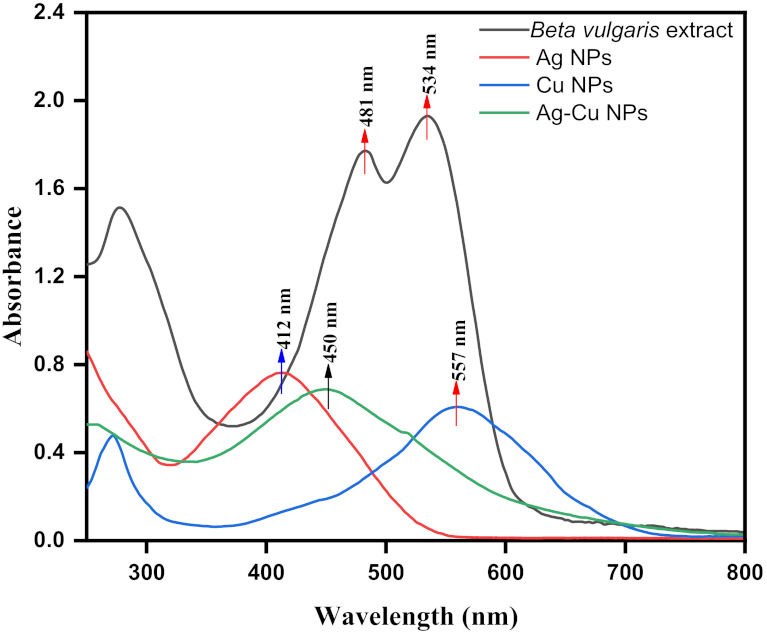
UV–Visible spectra of *Beta vulgaris* extract, Ag NPs, CuNPs and Ag-Cu bimetallic nanoparticles.

UV–Vis spectra after the measurement of optical properties were inferred for the biosynthesis of Ag-Cu nanoalloy from *Beta vulgaris* L. extract, as shown in Figure 3. *Beta vulgaris* L. extract is a mixture of several phytochemicals and pigments that showed characteristic absorption peaks intensities at λ_max_ ca. 481 nm and ca. 534 nm corresponding to yellow betaxanthin and red-purple betanin, respectively, [53,54]. Meanwhile, an absorption intensity peak at ca. 412 nm was observed from Ag NPs, and Cu NPs showed characteristic peak intensity at ca. 557 nm. The observed local surface plasmon resonance (LSPR) showed the effect of Cu fabrication onto surface of Ag NPs in biosynthesized Ag-Cu nanoalloy as reflected from the absorption peaks observed in UV–Visible spectroscopy. This fact can be viewed after their redox potentials, as the higher redox potential of Ag^+^ to Ag (0.799 V) compared with Cu^2+^ to Cu (0.34 V) emphasizes the Cu particles are not possible formed before the reduction of all Ag+ in the reaction mixture [55]. We observed a lower shift of peak at λ_max_ ca. 481 nm and peak at λ_max_ ca. 557 nm of *Beta vulgaris* extract and Cu NPs, respectively. Also, the higher shift of peak at λ_max_ ca. 412 nm of Ag NPs accentuates Ag-Cu nanoalloy’s biosynthesis as inferred from peak intensity at λ_max_ ca. 450 nm. However, the obtained results of the spectrum emphasized the biosynthesis of Cu nanoparticles onto the surface of Ag nanoparticles and was enhanced during the one-pot biosynthesis Ag-Cu nanoalloy from *Beta vulgaris* extract with alternate blue and redshift in the absorption band.

### 3.2. FTIR Spectra of Beta vulgaris L. Extract Assisted Ag-Cu Bimetallic Nanoalloys

**Figure 4:**
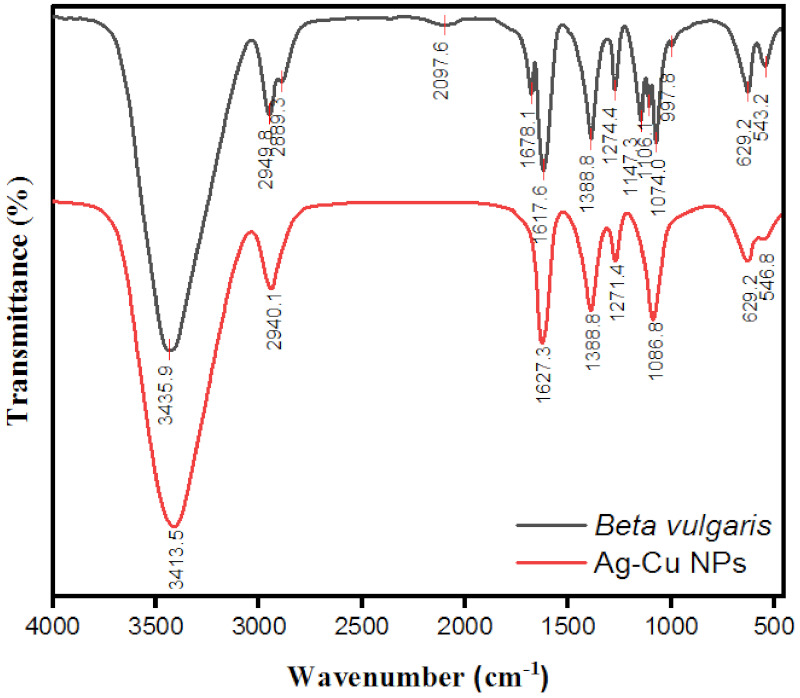
Fourier transforms infrared (FTIR) spectra of *Beta vulgaris* extract and Ag-Cu bimetallic nanoparticles.

The FTIR analysis determines the role of various functional groups of phytochemicals, including pigments of *Beta vulgaris* L. extract involved in stabilizing and reducing metal ions during the biosynthesis of Ag-Cu nanoalloy. The observed various functional groups are possible as the *Beta vulgaris* L. extract possesses various phytochemicals such as compounds including Betanin, Isobetanin, Vulgaxanthin I and II Flanovids, phenolic amides, and phenolic acids [56]. The FTIR analysis was advanced to deduce the possible mechanism of metal reduction and betanin within *Beta vulgaris* L. extract. The FTIR spectrum, as depicted in Figure 4, shows major peak intensities across a transmission range of 3600–3200 cm^−1^, i.e., ν ~ 3435.9 cm^−1^ attributed towards the phenolic hydroxyl (–OH) stretching vibrations. The presence of phenolic hydroxyl –OH suggests proton dissociation from –OH group could be accompanied by reduction of the metal ion. The observed peaks between ν ~ 3000–2850 cm^−1^, i.e., 2949.8 cm^−1^ was attributed to the presence of C–H stretching of alkanes. The observed frequencies between ν ~ 1680–1620 cm^−1^, i.e., 1678.1 cm^−1^ and 1617.6 cm^−1^ are due to –C=C stretching vibration of alkenes. FTIR frequencies between v ~ 1200–1800 cm^−1^ i.e., 1388.8 cm^−1^ and 1274.4 cm^−1^ are corresponding stretching vibration frequencies exhibited by carbonyl groups (–C=O). The presence of these molecular groups provides stability and capping to biosynthesized bimetallic nanoalloys. In the frequency range between ν ~ 1250–1080 cm^−1^, i.e., 1147.3 cm^−1^ and 1106.1 cm^−1^ was attributed to the presence of (C–N) stretching vibration of aliphatic amines viz. due to proteins. In addition, the symmetric and asymmetric C–O–C stretching appeared in the frequency range of ν ~ 1016–1060 cm^−1^, i.e., 1074 cm^−1^ [57]. The previously reported, decreased bands observed in monometallic Ag NPs, were between v ~ 621.1 and 406.09 cm^−1^ as facilitated by betanin [4,58]. Similarly, the decreased frequencies v ~ 629.2 cm^−1^ and v ~ 543.2 cm^−1^ are observed after the biosynthesis of bimetallic Ag-Cu nanoalloy facilitated by betanin. However, the possible mechanism behind the biosynthesis of Ag-Cu nanoalloy was suggested to be a reduction reaction between betanin of *Beta vulgaris* L. extract and metal ions of the reaction mixture.

### 3.3. XRD Analysis of Beta vulgaris L. Extract Assisted Ag-Cu Bimetallic Nanoalloys

**Figure 5:**
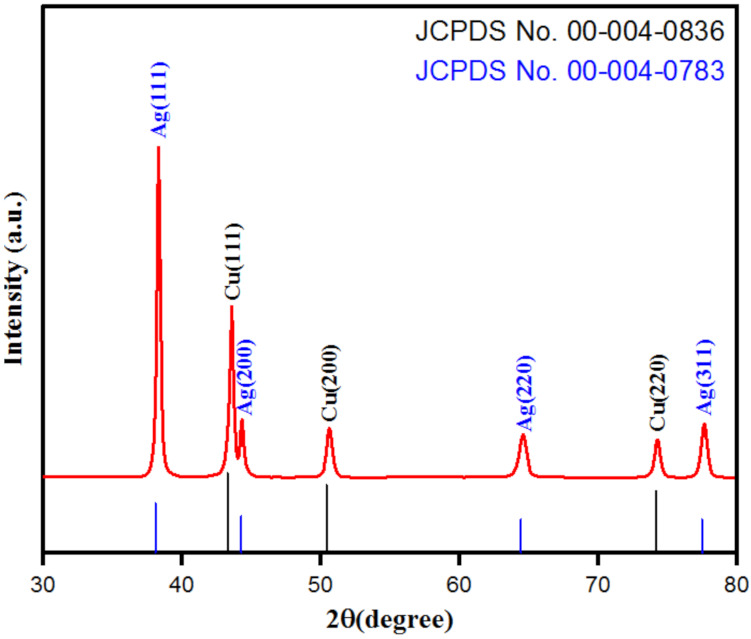
X-ray diffraction (XRD) pattern of *Beta vulgaris* assisted Ag-Cu bimetallic nanoparticles.

The X-ray diffraction (XRD) patterns of biosynthesized Ag-Cu nanoalloy from *Beta vulgaris* L. extract were depicted in Figure 5. The occurrences of the diffraction pattern in bimetallic nanoparticles with sharp peaks deduce their crystalline structures [59]. In biosynthesized Ag-Cu nanoalloy, obtained diffraction peaks at angle 2θ with corresponding diffraction patterns emphasized Cu’s face-centered cubic (fcc) phase with Ag crystals. Additionally, from close perusal of Figure 5, the metallic silver (JCPD card No. 004-0783) was detected from recorded diffractions peaks at 38.5, 44.8, 64.7, and 77.5 with corresponding crystallographic planes (111), (200), (220) and (311), respectively, specifying the standard fcc structures of silver particles [60]. Moreover, the presence of Cu nanoparticles was deduced from the 2θ angle at 43.8, 50.4 and 73.6 with alternate crystallographic planes (111), (200), (220) and (311), respectively, from metallic Cu (JCPD card No. 00-004-0836) corresponding to the fcc planer phase of Cu nanoparticles with Ag nanoparticles in a bimetallic Ag-Cu nanoalloy [61]. However, the Scherrer equation was utilized to calculate the average crystalline size of biosynthesized Ag-Cu nanoparticles at intense peak at angle 2θ. Average crystalline size with highest size distribution was calculated as per Scherrer equation (d = Kλ/βcosθ) and was found 20.29 nm. The different parameters of Scherrer equation such d represent the crystallite size, K as Scherer constant equal to 0.9, λ to the wavelength of X-ray sources (typically 1.5406 Å), β relays on FWHM in radians, and θ signify the peak position (Bragg angle). Furthermore, the obtained crystalline size of nanoalloy was conformed from the SEM analysis and was found almost similar in numerical values.

### 3.4. SEM, EDX, and TEM of Beta vulgaris L. Assisted Synthesis Ag-Cu Bimetallic Nanoalloys

**Figure 6:**
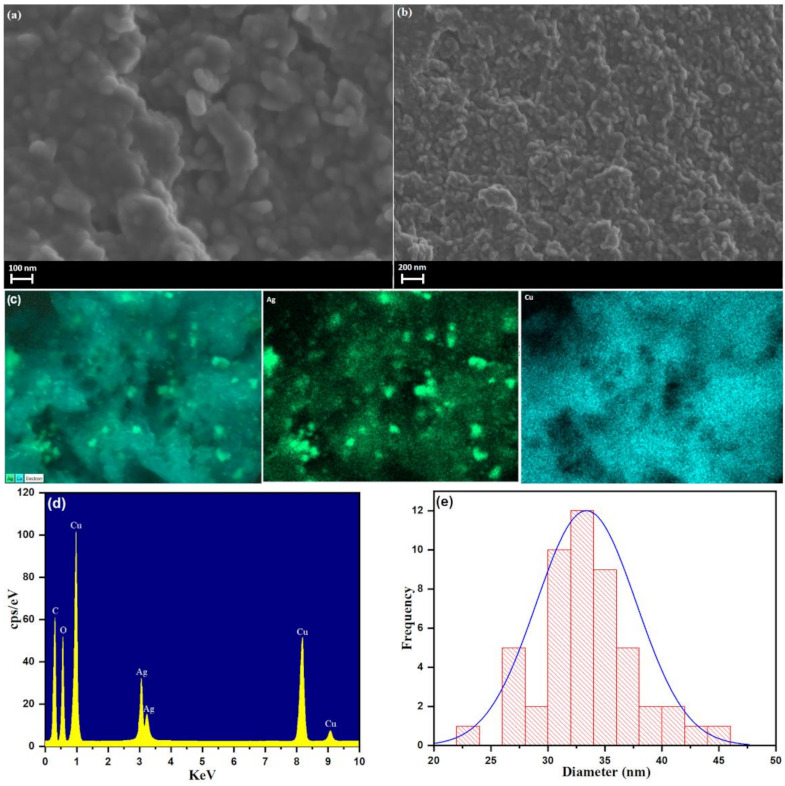
(**a**,**b**) Scanning electron microscope (SEM) images, (**c**) elemental mapping, (**d**) energy-dispersive X-ray spectra (EDX), and (**e**) particle size distribution of Ag-Cu bimetallic nanoparticles.

**Figure 7:**
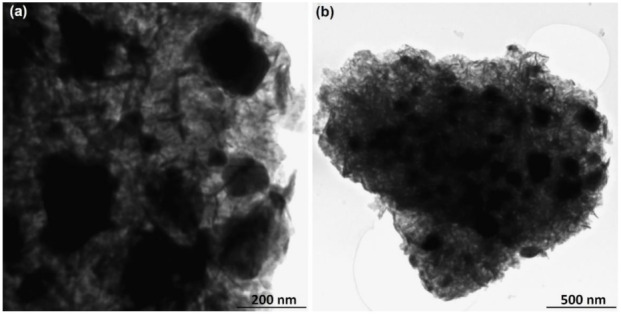
Transmission electron microscope (TEM) images of Ag-Cu bimetallic nanoparticles at different magnifications. (**a**) 200 nm and (**b**) 500 nm.

The SEM analysis was used to justify the obtained morphology and structure of biosynthesized Ag-Cu nanoalloy, as depicted in Figure 6a,b. The semi-spherical agglomerated bimetallic Ag-Cu clusters are prominently observed at 200 nm resolution in Figure 6a,b. However, under the high resolution of about 100 nm, bimetallic Ag-Cu nanoalloy retains a nanostructure of silver and copper in semispherical morphology and these nanoparticles are fused to each other, as illustrated in Figure 6a,b. For Ag-Cu nanoparticles, more detailed elemental mapping was carried out using higher magnification, as shown in Figure 6c. The Cu nanoparticles have a distinct predomination over the Ag nanoparticles, as shown in the picture. Considering the magnification used, all mappings of bimetallic samples show a regular spread of Ag and Cu. In addition, the EDX elemental analysis was performed to determine the elemental composition of the bimetallic Ag-Cu nanoalloy, as shown in Figure 6d. The EDX elemental detection also confirms the purity of the synthesized Ag-Cu nanoparticles. The observed Ag high-intensity peak compared to Cu correlates to the presence of crystalline structure in biosynthesized Ag-Cu nanoalloy. Besides, the biogenesis of Ag-Cu nanoalloy was realized from the lower eV positions of corresponding C and O atoms. Moreover, Figure 6e depicts the size distribution histogram of Ag-Cu NPs. The values for particle size are represented as the mean diameter (d_mean_) and image software was used to calculate an average particle size in bimetallic Ag-Cu nanoalloy of about 33 nm. Furthermore, the bimetallic Ag-Cu nanoalloy was characterized by TEM analysis at different magnification power, as shown in Figure 7a,b. The TEM micrographs of bimetallic Ag-Cu NPs show semispherical morphology with different sizes. These bimetallic Ag-Cu nanoparticles were found to be agglomerated and incorporated in a dense, thick pattern on the transmission electron microscopy pictures, suggesting that they may be serving as stabilizing chemical ingredients in the aqueous extract of *Beta vulgaris*.

### 3.5. TGA-DTG Curve of Beta vulgaris L. Assisted Synthesis Ag-Cu Bimetallic Nanoalloys

**Figure 8:**
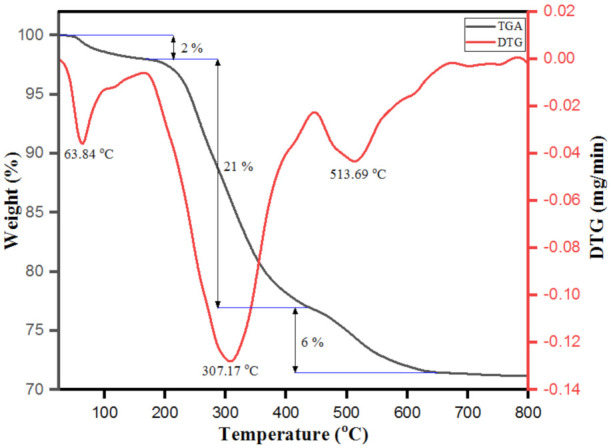
Thermogravimetric analysis (TGA) and derivative thermogravimetric (DTG) curves of Ag-Cu bimetallic nanoparticles.

The TGA/DTG, thermal stability, and the thermal degradation patterns are analyzed to comprehend the role played by phytochemicals of *Beta vulgaris* L. extract as capping/stabilizing agents onto the surface of biosynthesized Ag-Cu nanoalloy, depicted in Figure 8. Thermogravimetric analysis of weight loss was operated under nitrogen atmosphere at a heating rate of 10 °C/min; the observed weight loss process was further explained in a stepwise manner from the TGA/DTG curves (Figure 8). The analyzed weight loss during the thermal decomposition observed from the degradation pattern of biosynthesized Ag-Cu nanoalloy was approximately 29% by weight. The close perusal of Figure 8 emphasizes that the regions of weight loss observed from the TGA analysis were in the ranges 0–270 °C, 270–410 °C, and 410–650 °C, respectively. The observed DTG peak at 63.84 °C in the region of TGA 0–270 °C (weight loss by 2%) was apparently from the loss of moisture content from the surroundings and the volatile phytochemicals adsorbed onto the surface of Ag-Cu nanoalloy. Additionally, the maximum weight loss in the TGA region 270–410 °C (about 21%) with DTG peak at 307.17 °C was believed from the thermal decomposition of biomolecules including coloring pigment such as betanin of *Beta vulgaris* L. extract, which acts as reducing agents during biosynthesis of bimetallic Ag-Cu nanoalloys. Moreover, the presence of other constituents, including phenolic acids, proteins, and flavonoids of *Beta vulgaris* L. extract as capping/stabilizing agents, were determined from the DTG peak at 513.69 °C in the region of TGA between 410–650 °C (with weight loss 6%). TGA/DTG curves obtained further approximate *Beta vulgaris* L. extract assisted biosynthesis of thermally stable Ag-Cu nanoalloy. However, in general, the thermally stable nanoparticles with a high ratio of surface area to volume possess an efficient capability to inhibit bacterial and fungal growth. Silver in its native metallic state is being inert while it gets ionized in contact with moisture. The ionized form of silver has been reported to be highly reactive in binding with bacterial cell walls, leading to denaturation of tissue proteins and structural changes with cell distortion and death [62]. Silver nanoparticles can deteriorate bacterial growth upon binding with DNA and RNA of bacterial cells with inhibition of bacterial replication [63,64]. Meanwhile, the Cu and CuO nanoparticles are reported to possess a broad spectrum of antimicrobial activities [65]. Based on the literature survey and keeping the inherent chemical properties of both mono-metals in mind, we assessed the development of Ag-Cu nanoalloy from *Beta vulgaris* L. extract and evaluated its applicability against the growth inhibition and virulence in *C. albicans*.

### 3.6. Antifungal Activity of Ag-Cu Bimetallic NPs

With the onset of drug-resistant *Candida* infections, this opportunistic fungus has gained much consideration in current years. However, nanobiotechnology has already been proven successful in treating such infections. The antifungal susceptibility showed that Ag-Cu bimetallic NPs possess potent anticandidal activity against different *C. albicans* strains (Table 1). As recorded, the MIC and MFC values against FLZ susceptible strains were in the range of 0.5–2.0 μg/mL and 2.0–8.0 μg/mL, respectively. However, the MIC and MFC values for FLZ resistant strains were in the range 8.0–16.0 μg/mL and 16.0–64.0 μg/mL, respectively. On the other hand, the MIC values for FLZ against susceptible and resistant strains were 0.25–0.5 μg/mL and 16.0–64.0 μg/mL, respectively.

Nanobiotechnology can provide ‘a way out’ for treating infections caused by multidrug-resistant pathogens [66]. Results in this study are consistent with the literature, where different Ag and Cu nanoparticles were reported to possess anti-*Candida* activity, even though the MIC and MFC values in this study are different from the other findings [31,67,68]. There are various explanations for variable antimicrobial properties of NPs, such as size and shape of NPs [69], the stabilizing agent used during synthesis [70], and also varies from species to species [71]. Additionally, it has been investigated whether nanoparticles display their antifungal activity by destabilizing fungal cell membrane mainly due to damaging membrane integrity, discharge of intracellular ions, and inhibiting normal budding process [72]. Another study concluded that silver NPs tend to inhibit various dermatophytes and *Candida* species [73]. Similar to Ag NPs, Cu NPs have also been good antifungal activity against *C. albicans* [74,75,76]. The other important factor of high antifungal activity of these bimetallic Ag-Cu NPs could be the synergistic combination of these metals in this complex. Therefore, these novels Ag-Cu NPs have enhanced activity against both drug-susceptible and drug-resistant fungal species.

### 3.7. Ag-Cu Bimetallic NPs Effect Cell Viability in C. albicans

**Figure 9:**
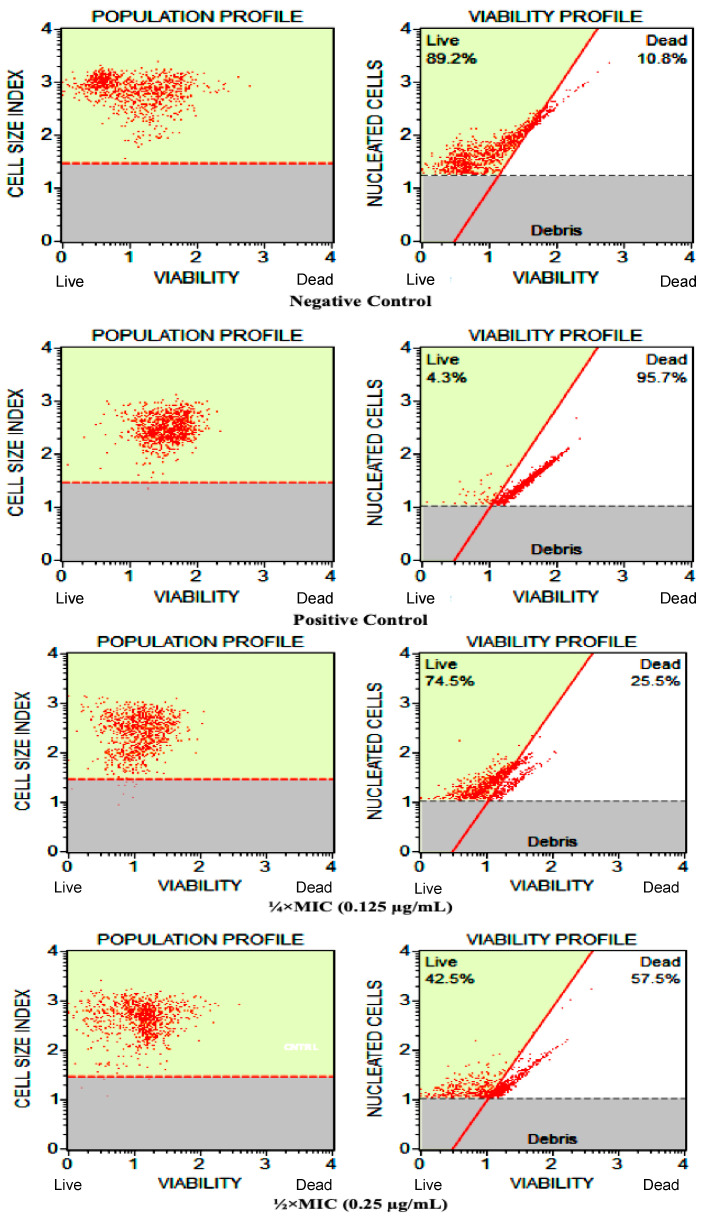
Effect of Ag-Cu bimetallic NPs on cell count and viability of *C. albicans* SC5314. Figure demonstrates viability and population profile of *C. albicans*. Healthy growing *C. albicans* represented as negative control; heat-killed cells as positive control; *C. albicans* exposed to Ag-Cu bimetallic NPs at different MIC values (¼ × MIC and ½ × MIC).

The effect of test NPs on the cell viability profile of *C. albicans* was tested at different concentrations (¼ × MIC and ½ × MIC), and the results are presented in Figure 9. Results indicated that the test NPs at sub-inhibitory concentrations considerably decrease the cellular viability compared to control cells. Post-analysis, it was observed that at ¼ × MIC value, there was 25.5% ± 2.7% cell death, while a significant increase in the percentage of cell death (57.5% ± 2.0%) was detected after the cells were exposed to ½ × MIC value. Unexposed and heat-killed cells were used as negative and positive controls, showing 10.8% ± 1% and 95.7% ± 2% dead cells, respectively. In the population profile square (Figure 9), debris was omitted from the cell population based on cell size index to maintain the assay’s accuracy and avoid false results. From the viability profiling of these nanoparticles, which reported the fungicidal activity of these bimetallic NPs, it can be concluded that these NPs could have targeted fungal cell membrane and thereby disrupt cellular integrity, which results in cell death. However, further studies are required to confirm these claims.

### 3.8. Ag-Cu Bimetallic NPs Impedes Adherence of C. albicans to Polystyrene Surface

**Figure 10:**
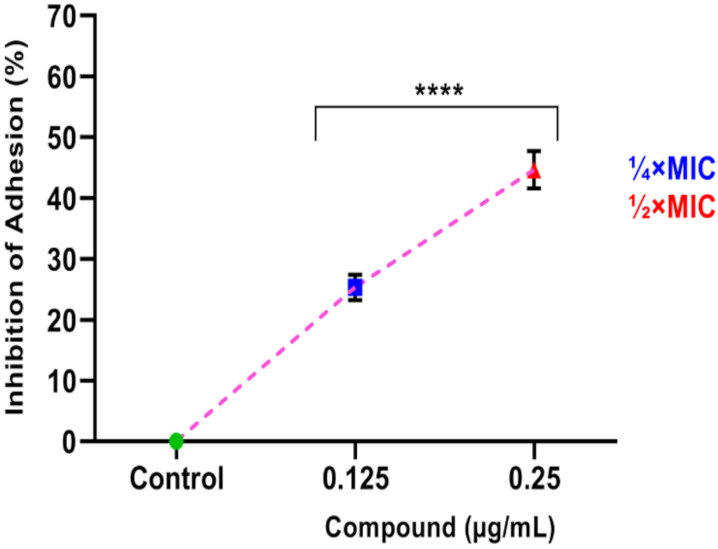
Effect of Ag-Cu bimetallic NPs on *C. albicans* adherence. Strain SC5314 cells were exposed to ¼ MIC and ½ MIC of the test NP. Untreated cells (Control) represent 0% inhibition of adherence whereas, the adherence was found hindered in exposed *C. albicans* cells. **** *p* value < 0.0001.

The effect of test NPs on adherence property of *C. albicans* was evaluated in 96-well microtiter plate. The results showed that the exposure of bimetallic NPs at subinhibitory concentrations strongly hindered the attachment capacity of yeast cells to the polystyrene plate in a dose-dependent manner (Figure 10). The percentage of inhibition was calculated as 29% ± 2% and 53.67% ± 3.5% at ¼ × MIC and ½ × MIC values, respectively. Therefore, the results demonstrated that test NP at subinhibitory concentrations significantly hampered the adherence phenomenon in *C. albicans*.

The application of Ag and Cu NPs to inhibit adherence of *Candida* to the denture surface for preventing oral candidiasis has been widely reported [77,78]. Additionally, NP strongly inhibits *Candida* adherence to denture surfaces because of high exposure of metal ions to these surfaces [78]. Therefore, in line with the previous discussion, the combination of two metals has a significant effect on the adhering property of *C. albicans.* Thus, further attempts should be made to confirm the usage of Ag-Cu bimetallic NPs for coating denture base materials for clinical use.

### 3.9. Ag-Cu Bimetallic NPs Inhibits Morphological Transition in C. albicans

**Figure 11:**
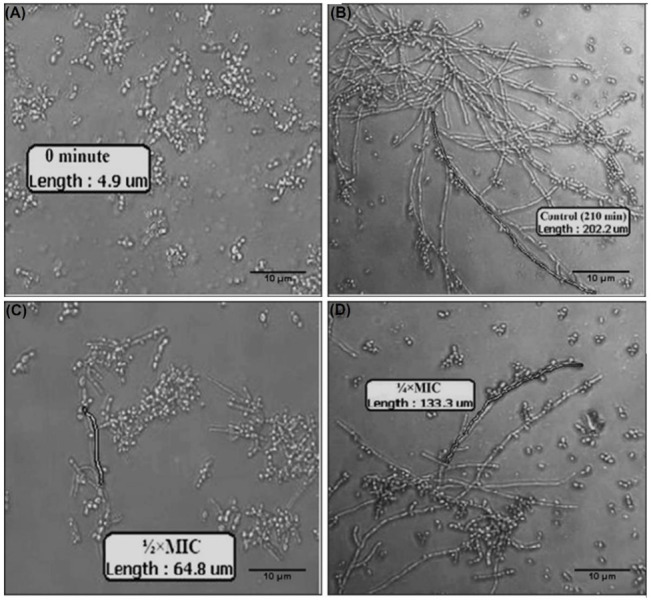
Inhibitory effect of bimetallic Ag-Cu NPs on yeast to hyphal transition in *C. albicans*. (**A**) Yeast cells recorded at 0 min; (**B**) Yeast to hyphae transition observed after growing *C. albicans* for 210 min in the absence of test NPs; Yeast to hyphae transition after 210 min in the presence of test NP at concentration 0.125 µg/mL (**C**) and 0.25 µg/mL (**D**).

The effect of Ag-Cu bimetallic NPs on *C. albicans* morphogenesis (yeast to hyphae transition) was visualized microscopically. The results revealed that the testing of NP at its subinhibitory concentrations obstructs the morphological change in *C. albicans*, whereas the unexposed control group underwent a morphogenesis phenomenon (Figure 11). The percentage of yeast to hyphae transition and the length of the hyphal form (scale of 10) in both test and control at different time intervals are summarized in Table 2. When compared, control yeast cells displayed 52–55% yeast to hyphae transition in 120 min, whereas only 6–9% transition was observed at ¼ × MIC value followed by complete hyphal inhibition at ½ × MIC. Furthermore, unexposed control cells showed almost 100% transition in 210 min. However, in the presence of the NPs, the population showing transition was markedly reduced; only 46–50% and 18–21% hyphal transition were detected at ¼ × MIC and ½ × MIC, respectively. Additionally, cells treated with test NP also demonstrated a significant consequence on their hyphal length compared to control. Readings were also noted after 30, 60, and 90 min but not shown as the results were almost similar to those of 0 min.

In *C. albicans,* yeast to hyphal transition is a vital virulence attribute, which helps invade host tissues and form biofilms [79]. Previous studies have reported that different NPs target the morphological transition of *Candida* species [67]. In a recent study, Ag nanoparticles have been seen inhibiting germ tube formation in *C. albicans* at a concentration of 0.25 mg/mL [67]. The results in this study are more promising, as the MIC values of the test bimetallic NPs are low, and the effect on the morphological transition was even observed at sub-MIC values, which can again be linked to the synergistic interactions of the two NPs in these bimetallic NPs. In an another study, differently synthesized Ag nanoparticles were reported to do the morphological alterations in *C. albicans*, which has been linked to the ROS generation by these nanoparticles in *Candida* cells [31]. The results in the current study can link up using the bimetallic NPs to target virulence in pathogenic fungi along with targeting the growth inhibition and cell death. This anti-morphogenetic property of these NPs also opens a new window for the prevention of *C. albicans* biofilm formation on medical devices as the two processes are directly linked to each other.

### 3.10. Ag-Cu Bimetallic NP Abrogates Biofilm Formation in C. albicans

**Figure 12:**
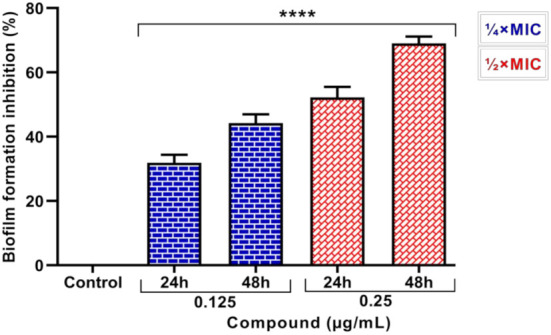
Effect of Ag-Cu bimetallic NPs on biofilm formation by *C. albicans*. The cells were treated with ¼ × MIC and ½ × MIC under biofilm growing conditions. Untreated cells (control) presents as 0% inhibition in biofilm formation, whereas inhibition was recorded in exposed cells. **** *p* value < 0.0001.

As morphological transition and adherence are directly linked to biofilm formation, the current study aimed to check the effects of newly synthesized bimetallic Ag-Cu NPs on biofilm formation. The results indicated that the test NPs significantly inhibit biofilm formation in *C. albicans* (Figure 12). At treatment of cells to ¼ × MIC, there was 31% ± 2% and 44% ± 1% inhibition in biofilm formation after 24 h and 48 h, respectively. These figures at ½ × MIC value of test NPs were 52% ± 1% and 69% ± 1% after 24 h and 48 h, respectively. Furthermore, the rate of inhibition in biofilm formation was directly dependent on time and concentration.

### 3.11. Ag-Cu Bimetallic NPs Detaches Mature C. albicans Biofilms

**Figure 13:**
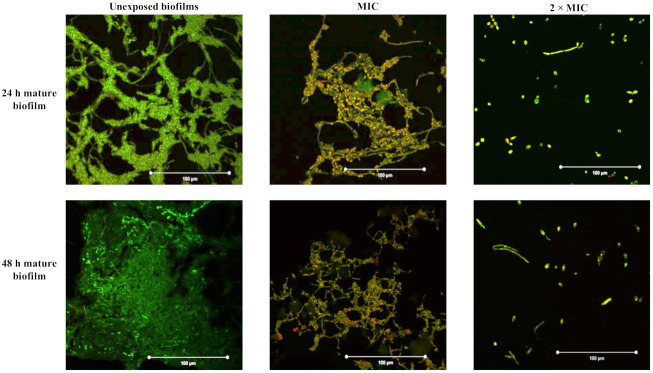
Inhibitory effect of Ag-Cu bimetallic NPs on mature *C. albicans* biofilms. The figure represents CLSM analysis of *C. albicans* SC5314 biofilms after exposure to test nanoparticles at various maturation phases (24 h and 48 h). The green fluoresces (Con A) represent the *C. albicans* cell wall and biofilm matrix; yellow-green fluoresces (FUN-1) represent dead/metabolically inactive cells.

The CLSM studies further confirmed the destructive ability of Ag-Cu bimetallic NPs. In the NP-free group, biofilms formed by *C. albicans* SC5314 possessed an atypical three-dimensional structure, composed mainly of proper hyphal structure. The biofilm also showed a well-defined and dense biofilm matrix with bright green fluorescence. Con A dye combines alpha-mannopyranosyl and alpha-glucopyranosyl residues present in the biofilm matrix. However, the structure of *C. albicans* biofilm was destroyed in the presence of Ag-Cu bimetallic NPs. At the MIC value of Ag-Cu NPs, the biofilm lacked true hyphal structure and was predominantly composed of pseudohyphae and yeast cells; whereas, when 2 × MIC of Ag-Cu NPs was added, the *C. albicans* biofilm showed an abrogated architecture composed mainly of yeast cells and lacking both actual hyphal and pseudohyphal structures. Furthermore, the cells showed altered viability, which is clearly represented by yellow-green fluorescence. In non-viable cells, FUN-1 dye remains confined to the cytosol and fluoresces yellow-green depicting metabolically inactive cells (Figure 13).

In *C. albicans* morphological transition is interlinked to biofilm formation [79]. *C. albicans* biofilms play a critical role in pathogenesis as these biofilms can firmly build on all types of medical devices [80]. The anti-biofilm property of Ag-Cu NPs can be valuable from a therapeutic point of view. In vitro results in the present study highlighted that these bimetallic NPs could block biofilm formation in *C. albicans* even at lower concentrations. Recently, Ag NPs have shown anti-biofilm activity against *C. albicans* [81,82]. Similarly, Cu NP is also well established for inhibiting biofilm formation in *C. albicans* [83]. The exact mechanism of action of these nanoparticles are not known. However, researcher’s proposed that the easy binding and higher infiltration of nanoparticles into the extracellular biofilm matrix results in biofilms destruction as well as their ability to inhibit yeast to hyphae transition, the viability of both yeast and hyphal forms and cell wall disruption may also lead to its anti-biofilm property [66,84]. Therefore, the anti-morphogenetic and anti-biofilm properties of these Ag-Cu bimetallic NPs emphasize the need to further study these NPs to develop novel antifungal drugs that will be beneficial in treating infections caused by drug-resistant pathogens.

Additionally, in mature biofilms, the extracellular matrix act as an obstruction for drug infiltration, and therefore, the cells impregnated within the biofilm matrix are more resistant to antifungal drugs even at higher concentrations (1000-fold higher than the planktonic IC_50_ values) and are therefore difficult to eradicate [85]. However, the present investigation proves that Ag-Cu NPs at a concentration of 0.5 µg/mL (MIC value) successfully detached the 24 h and 48 h mature biofilms, resulting in scattered hyphal structures composed of pseudohyphae and yeast cells. Furthermore, a concentration of 1.0 µg/mL (2 × MIC) was responsible for destroying mature biofilm, blocked ex-exopolysaccharides’ production, and altered cell viability. In comparison, the unexposed mature biofilms showed the intense network of yeasts, hyphal and pseudohyphal forms embedded into a complex three-dimensional structure. Lara and co-workers have well established the anti-biofilm potency of silver nanoparticles, and their observations are similar to the present study [66]. They also reported a dense and well-defined network of yeast and hyphae with an abundant extracellular polymeric substance. In addition, the biofilm showed scarce appearance, wall damage, and compromised viability after treatment with silver nanoparticles.

Therefore, this result verifies that Ag-Cu bimetallic NPs substantially affect the integrity of cells within the biofilm matrix, indicating its capability to breach the biofilm matrix, thereby attacking the crucial virulence attribute of *C. albicans*.

### 3.12. Ag-Cu Bimetallic NPs Reduces Hydrolytic Enzymes Production in C. albicans

**Figure 14:**
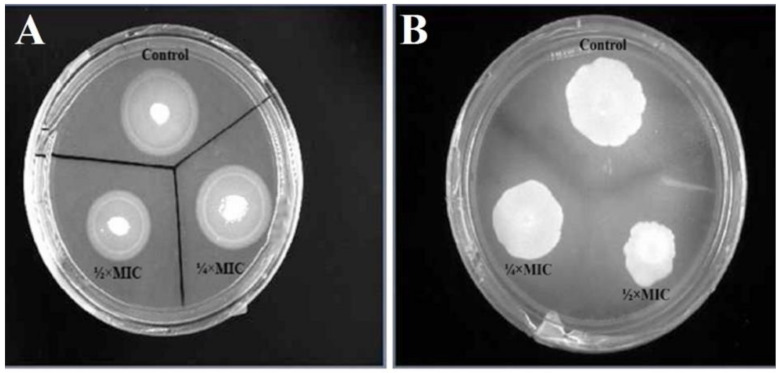
Representative plates of secretion of extracellular hydrolytic enzymes. (**A**) represents proteinase plate and (**B**) represents phospholipase. Untreated cells with turbid colonies surrounded by zone of protein degradation (**A**) and zone of lipid precipitation (**B**) serves as control, whereas other 2 zones represent cells exposed to ¼ MIC and ½ MIC bimetallic Ag-Cu NPs.

**Figure 15:**
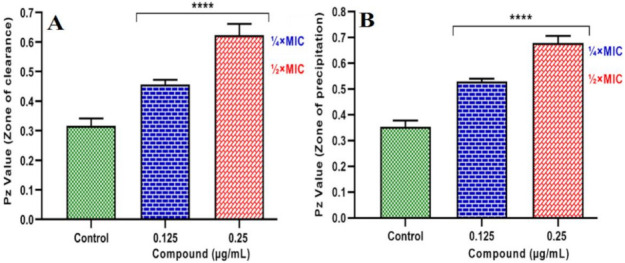
Effect of bimetallic Ag-Cu NPs on proteinase (**A**) and phospholipase (**B**) production by *C. albicans*: The yeast cells were exposed to ¼ × MIC and ½ × MIC values of bimetallic Ag-Cu NPs. The untreated cells are represented as control. A high Pz value represents low enzyme activity. Data are presented from three independent experiments using means ± S.D. **** *p* value < 0.0001.

The present investigation revealed that bimetallic NP at different concentrations (¼ × MIC and ½ × MIC) retards the production of extracellular proteinases and phospholipases in *C. albicans*. According to the obtained results, there was a reduction in the secretion of enzymes in the exposed *C. albicans* cells compared to their respective controls. Figure 14A, clearly demonstrates the proteinase assay in untreated and NPs treated cells, and the precipitation zones produced are visible. In contrast, the results of proteinase enzyme activity have been summarized in Figure 15A. When calculated, it was observed that at ¼ × MIC and ½ × MIC values, the inhibition of proteinase secretion was 44% ± 2% and 93% ± 1%, respectively.

Furthermore, results also demonstrated a significant inhibitory effect of bimetallic NP on phospholipase secretion. The opaque zones produced can be seen in Figure 14B, and the results were summarized in Figure 15B. When analyzed quantitatively, at ¼ × MIC and ½ × MIC values, there was 51% ± 3% and 94% ± 2% inhibition in phospholipase secretion, respectively.

The extracellular hydrolytic enzymes are an essential attribute for *Candida* pathogenicity. Enzymes such as proteinase and phospholipase play a key role in establishing *Candida* species infection in human hosts [86]. Thus, intervention in these pathogenic attributes has appeared as a novel drug target for upcoming molecules. Despite playing an essential role in pathogenicity, there are minimal studies reporting the anti-virulent properties of nanoparticles against *Candida*, and no study reporting the effect and mechanism of Ag/Cu NPs against these hydrolytic enzymes [67,87,88]. In the present study, the inhibitory property of bimetallic NPs on the secretion of proteinase and phospholipase from *C. albicans* was demonstrated for the first time. Besides playing an essential role in pathogenicity, these enzymes are linked to adhesion, germ tube formation, yeast to hyphae transition, infiltration, and host tissue injuries [89]. The extracellular proteinases are also known for degrading various human proteins on the infection site, tissue invasion, and penetration [90]. This study observed that bimetallic Ag-Cu NPs at 0.25 µg/mL drastically lowered the proteinase and phospholipases activity by 93% and 94%, respectively, in *C. albicans,* and dose-dependent inhibition. Previously researchers have also reported comparable results [67,87,88], therefore supporting results from this study that bimetallic Ag-Cu NPs can be explored to treat stubborn *Candida* infections.

### 3.13. Bimetallic Ag-Cu NPs Downregulates Pathogenicity Associated Genes in C. albicans

**Figure 16:**
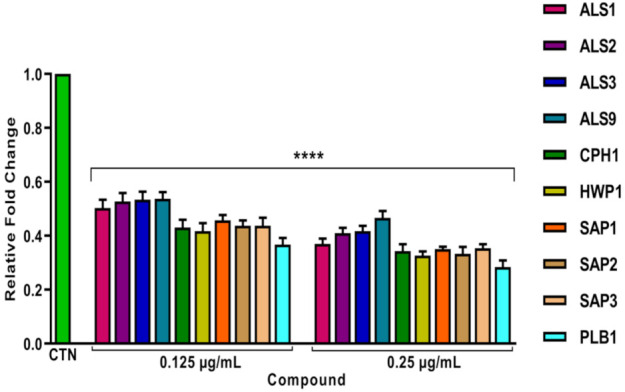
Effect of bimetallic Ag-Cu NPs at subinhibitory concentrations (¼ × MIC and ½ × MIC) on the expression of genes involved in pathogenicity in *C. albicans* SC5314. Relative gene expression was calculated after normalization to housekeeping genes (*ACT1*, *PMA1,* and *RPP2B*). Unexposed cells (CTN) were considered as a negative control. **** *p* value < 0.0001.

The bimetallic NP was found to downregulate the expression of pathogenicity-related genes in *C. albicans* (Figure 16). The expression levels of designated genes were given as relative values compared to the untreated cells (set as 1.0). Therefore, gene expressions of *ALS1*, *ALS2*, *ALS3* and *ALS9* (adherence), *CPH1*, *HWP1* (morphogenesis), *SAP1*, *SAP2*, and *SAP3* (proteinases) and *PLB1* (phospholipases) were significantly downregulated after exposure to subinhibitory concentrations (¼ × MIC and ½ × MIC) when compared to control cells. The expression level recorded at ¼×MIC value for genes related to adherence, morphogenesis, proteinases, and phospholipases were significantly downregulated with a fold change of 1.8–2.0, 2.3–2.4, 2.1–2.3 and 2.7, respectively. These figures at ½ × MIC value ranged from 2.1–2.7, 2.9–3.0, 2.9–3.0, and 3.6 folds, respectively.

The gene expression profile of significant pathogenicity-related genes was investigated to elucidate the possible molecular mechanism displayed by Ag-Cu bimetallic NPs on *C. albicans*. In-depth analysis showed that Ag-Cu bimetallic NPs at sub-inhibitory concentrations downregulated expression of pathogenic related genes in *C. albicans*. The *ALS* gene family encodes for cell surface glycoproteins, which play a significant role in the adhesion of *C. albicans* to different surfaces in the host [91,92]. Therefore, downregulation of these genes could be responsible for inhibiting in vitro adherence and biofilm formation in exposed *C. albicans* cells. Furthermore, yeast to hyphal transition is considered a critical virulence attribute of *C. albicans*. *HWP1* is one of the most important hyphal regulons encoding hypha-specific cell wall proteins responsible for tissue penetration in the host [93], and *CPH* are hyphal-specific genes and triggers hyphal formation [94]. In present results, Ag-Cu bimetallic NPs have downregulated both hyphal-specific genes resulting in reduced hyphal growth and suppression of filamentation, which can directly impact adhesion and biofilm formation and detachment of mature biofilms.

Similarly, in *C. albicans*, SAPs, also known as sap protein family (mainly Sap1–Sap10) are well recognized for active penetration in host tissues, stabilizing cell wall integrity, and attachment of *C. albicans* with host cells and therefore considered as an essential virulence factor [95,96]. In contrast, *PLB*1 encodes phospholipases and triggers morphological transition, colonization, and cytotoxicity [97]. In-depth analysis showed that Ag-Cu bimetallic NPs also inhibited the expression of genes responsible for the production of extracellular enzymes in *C. albicans* and, therefore, decreased virulence intensity in *C. albicans*.

### 3.14. Cytotoxic Effect of Ag-Cu Bimetallic NP against Horse RBCs

**Figure 17:**
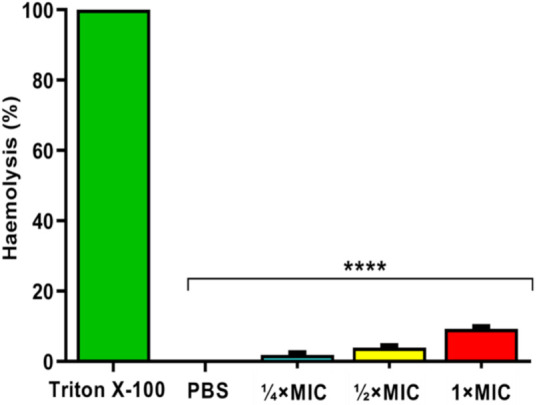
Haemolytic activity of Ag-Cu bimetallic NP at different concentrations. Haemolysis was determined by an absorbance reading at 450 nm and compared to haemolysis achieved with 1% Triton X-100 (reference for 100% haemolysis). PBS and 1% Triton X-100 served as negative and positive controls, respectively. **** *p* value < 0.0001.

The cytotoxicity at various concentrations (¼ × MIC, ½ × MIC and MIC) was evaluated in the present study (Figure 17). Triton X-100 resulting in 100% lysis serve as a positive control, and PBS showing no lysis of RBCs serve as a negative control. Ag-Cu bimetallic NP at ¼ × MIC, ½ × MIC and MIC values exhibited 1.8% ± 1.0%, 4.0% ± 2.0% and 9.5% ± 3.5% cell haemolysis, respectively. The results confirmed that the Ag-Cu bimetallic NP within its MIC ranges showed a significantly low cytotoxicity effect compared to the positive control, which causes 100% cell lysis and thereby recommends the possibly safe use of these NP.

The low cytotoxicity of Ag-Cu bimetallic NPs towards horse erythrocytes is attributed to the several modification processes involved during different steps of pre-and post-synthesis. In addition, it has been reported that the shape, size, and structure of NPs are the determining factors for their cytotoxicity. Therefore, stability and biocompatibility should be taken into consideration during synthesis [98]. However, in vivo studies will further advocate the safe use of Ag-Cu bimetallic NPs.

## 4. Conclusions

In the current study, it was demonstrated that Ag-Cu bimetallic NPs strongly inhibit the growth, viability, adherence, yeast to hyphae conversion, secretion of extracellular hydrolytic enzymes, and most importantly, formation of biofilm by *C. albicans*. The present finding suggests that Ag-Cu bimetallic NPs can be used as a promising therapeutic option for treating *Candida* infection by abrogating the critical pathogenic attributes. Coating urinary catheters and medical devices with Ag-Cu bimetallic NPs can also prevent biofilm-associated infections in immunocompromised patients. Moreover, this research work unwraps several other possibilities for the future study where further investigation of the molecular mechanisms involved in inhibition of virulence factors in *Candida* species can be undertaken. To reach biomedical applications, in vivo studies, biocompatibility analysis, and the safety profile of Ag-Cu bimetallic NPs need to be evaluated.

## Figures and Tables

**Table 1:** MIC and MFC values of Ag-Cu bimetallic NPs against different strains of *C. albicans*.

*C. albicans* Strains		Ag-Cu Bimetallic NPs (µg/mL)		FLZ (µg/mL)
		MIC	MFC	MIC
Control	SC5314	0.5	2.0	0.25
FLZ susceptible	4554	1.0	4.0	0.25
	4251	2.0	8.0	0.5
	4175	0.5	2.0	0.25
	4180	1.0	4.0	0.25
FLZ resistant	4324	8.0	32.0	32.0
	4106	8.0	16.0	32.0
	5112	16.0	64.0	64.0
	4085	8.0	16.0	16.0

**Table 2:** Yeast to hyphal transition in the presence of Ag-Cu bimetallic NP.

Time (min)	Control		¼ MIC		½ MIC	
	I	II	I	II	I	II
0	NG	0.244	NG	NG	NG	NG
120	52–55%	6.816	6–9%	1.443	NG	NG
150	77–80%	8.309	15–18%	2.647	2–5%	0.578
180	91–94%	8.956	29–32%	3.861	7–10%	1.374
210	97–100%	10	46–50%	6.632	18–21%	3.224

Note: NG, no hyphal growth; I—%-cells forming hyphae; II—length of hyphae on a scale of 10 µm.

## Data Availability

All relevant data are within the manuscript.

## Supplementary Materials

The following are available online at https://www.mdpi.com/article/10.3390/pharmaceutics13111957/s1, Table S1: List of primers used for RT-qPCR experiments.
