# Supplementary material for: Beta vulgaris Assisted Fabrication of Novel Ag-Cu Bimetallic Nanoparticles for Growth Inhibition and Virulence in Candida albicans
Source: Pharmaceutics. 2021 Nov 18;13(11):1957. doi: 10.3390/pharmaceutics13111957 (PMC8621205; doi:10.3390/pharmaceutics13111957)
Supplement: Supplementary file 1 [file pharmaceutics-13-01957-s001.zip › pharmaceutics-1452040-supplementary.pdf]

pharmaceutics

Supplementary Materials: *Beta Vulgaris* Assisted Fabrication of Novel Ag-Cu Bimetallic Nanoparticles for Growth Inhibition and Virulence in *Candida Albicans*

Majid Rasool Kamli, Maqsood Ahmad Malik, Shabir Ahmad Lone, Jamal S.M. Sabir, Ehab H. Mattar and Aijaz Ahmad

Table S1. List of primers used for RT-qPCR experiments.

Gene	Primer	Sequence (5′–3′)
ALS1	Forward	GCT CCA TCA CCT GCT GTT TC
	Reverse	CTG AGG TGC CTG TTG TCA AG
ALS2	Forward	TTT AAG GCT GGC ACC AAC AC
	Reverse	ATT GTG AAC CCC ATT GCA CC
ALS3	Forward	CTA CCG CTG TGA CCA CCT TA
	Reverse	CAG TTT CCC CAA TTG GTG CA
ALS9	Forward	CGA TTT CAG TTA GCA CCG CA
	Reverse	CGT TGA AGT TGG CAC CTC TC
CPH1	Forward	GTC GCC ACC CCA ACC TAT AT
	Reverse	AGG AAA CCC AGA AGC GTC AT
HWP1	Forward	CTG AAC CTT CCC CAG TTG CT
	Reverse	CGA CAG CAC TAG ATT CCG GA
SAP1	Forward	TTT GGT GGG GTT GAC AAA GC
	Reverse	ATG ACC TTG ACC GTC CAG TT
SAP2	Forward	CCG TTG GAT TTG GTG GTG TT
	Reverse	AGC ATT ATC AAC CCC ACC GA
SAP3	Forward	TGG TCC CCA AGG TGA AAT CA
	Reverse	TGT CCT TGA CCA GCT TGA CA
PLB1	Forward	ATA TGC TCC TGG TCC GGT TT
	Reverse	ATT GCT CTA TAC CCT CCG CC
ACT1	Forward	TGG TGA TGA AGC CCA ATC CA
	Reverse	CAT TGG AGC TTC GGT CAA CA
PMA1	Forward	GAA GGT GCT ACT GAT GCT GC
	Reverse	GCA ACA TCA GCG AAA ATG GC
RPP2B	Forward	ACA CCT CTC CAT CAG CTT CT
	Reverse	TGG GAC AGA AGC TAA TTT GGT G
